# Black African men with early type 2 diabetes have similar muscle, liver and adipose tissue insulin sensitivity to white European men despite lower visceral fat

**DOI:** 10.1007/s00125-019-4820-6

**Published:** 2019-02-06

**Authors:** Oluwatoyosi Bello, Cynthia Mohandas, Fariba Shojee-Moradie, Nicola Jackson, Olah Hakim, K. George M. M. Alberti, Janet L. Peacock, A. Margot Umpleby, Stephanie A. Amiel, Louise M. Goff

**Affiliations:** 1 Department of Diabetes, School of Life Course Sciences, Faculty of Life Sciences & Medicine, King’s College London, Franklin-Wilkins Building, Waterloo Campus, London, SE1 9NH UK; 2 Faculty of Health and Medical Sciences, University of Surrey, Guildford, UK; 3 School of Population Health and Environmental Sciences, King’s College London, London, UK

**Keywords:** Adipose insulin sensitivity, Black African, Ethnicity, Hepatic insulin sensitivity, Insulin sensitivity, Isotope, Lipolysis, Skeletal muscle insulin sensitivity, Tracer, Type 2 diabetes, Visceral fat

## Abstract

**Aims/hypothesis:**

Type 2 diabetes is more prevalent in black African than white European populations although, paradoxically, black African individuals present with lower levels of visceral fat, which has a known association with insulin resistance. Insulin resistance occurs at a tissue-specific level; however, no study has simultaneously compared whole body, skeletal muscle, hepatic and adipose tissue insulin sensitivity between black and white men. We hypothesised that, in those with early type 2 diabetes, black (West) African men (BAM) have greater hepatic and adipose tissue insulin sensitivity, compared with white European men (WEM), because of their reduced visceral fat.

**Methods:**

Eighteen BAM and 15 WEM with type 2 diabetes underwent a two-stage hyperinsulinaemic–euglycaemic clamp with stable glucose and glycerol isotope tracers to assess tissue-specific insulin sensitivity and a magnetic resonance imaging scan to assess body composition.

**Results:**

We found no ethnic differences in whole body, skeletal muscle, hepatic or adipose tissue insulin sensitivity between BAM and WEM. This finding occurred in the presence of lower visceral fat in BAM (3.72 vs 5.68 kg [mean difference −1.96, 95% CI −3.30, 0.62]; *p* = 0.01). There was an association between skeletal muscle and adipose tissue insulin sensitivity in WEM that was not present in BAM (*r* = 0.78, *p* < 0.01 vs *r* = 0.25 *p* = 0.37).

**Conclusions/interpretation:**

Our data suggest that in type 2 diabetes there are no ethnic differences in whole body, skeletal muscle, hepatic and adipose tissue insulin sensitivity between black and white men, despite differences in visceral adipose tissue, and that impaired lipolysis may not be contributing to skeletal muscle insulin resistance in men of black African ethnicity.

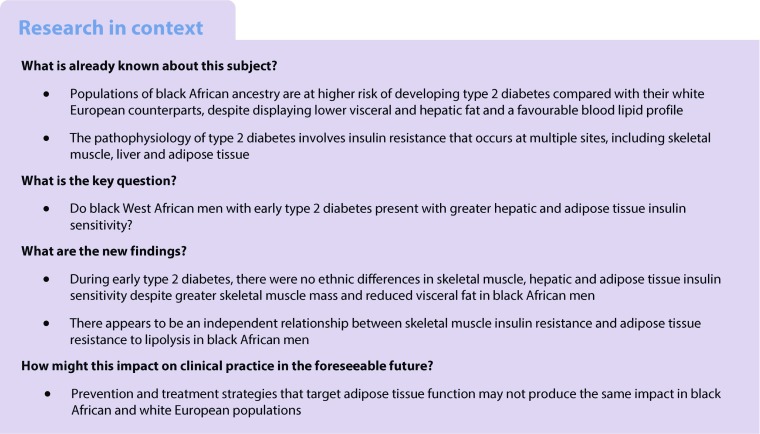



## Introduction

Populations of black African ancestry are disproportionately affected by type 2 diabetes compared with white Europeans [1]. The pathophysiological processes of type 2 diabetes are well documented and include beta cell dysfunction, ectopic fat deposition and insulin resistance of the liver, skeletal muscle and adipose tissue [2, 3]; the use of stable isotopes has enabled measurement of these tissue-specific sites of insulin resistance [4]. Black populations typically display lower visceral adipose tissue (VAT) and hepatic fat deposition and a more favourable blood lipid profile [5]. Visceral fat has been positively associated with hepatic and adipose tissue insulin resistance in diabetes and normal glucose tolerance [6]. The lower VAT exhibited in black populations suggests there may be ethnic distinctions in the pathophysiology of type 2 diabetes. There have been several studies comparing tissue-specific insulin resistance in vivo, using the ‘gold-standard’ hyperinsulinaemic–euglycaemic clamp and stable isotopes, in healthy populations of black and white ethnicities. These have been conducted primarily, although not exclusively, in women and adolescents but have not produced a consistent picture [7–20].

Sex differences in body composition have shown women to express greater central and overall body fat [21]. Accordingly, a sex distinction in the type 2 diabetes phenotype has been shown in black Africans, whereby men display greater insulin sensitivity compared with women [22]. The inconsistences in the findings from adolescent and female populations likely stem from differences in methods and participant body composition. Ethnic comparisons in people with type 2 diabetes are required to inform therapeutic decisions; however, they are also limited to adolescent and female populations [23, 24]. Peripheral insulin-stimulated glucose disposal has been shown to be similar in diabetic adolescents [23] but, to date, no study has compared this in diabetic black and white adults using stable isotope methods. Studies assessing ethnic differences in hepatic insulin sensitivity in type 2 diabetes are few; studies of basal endogenous glucose production have shown no difference in adolescents [23], but no studies have assessed insulin-stimulated suppression of endogenous glucose production and there have been no studies performed in adults with type 2 diabetes. In vivo assessment of adipose tissue insulin sensitivity in type 2 diabetes has been more limited*;* lower basal NEFA release has been reported in black women [24]; however, no study has assessed insulin-stimulated suppression of NEFA release. To date, no single study has undertaken a comprehensive ethnic comparison of whole body, peripheral, hepatic and adipose tissue sensitivity to insulin using the same study cohort and method.

We aimed to compare tissue-specific sites of insulin sensitivity between black (West) African men (BAM) and white European men (WEM) with early type 2 diabetes using the hyperinsulinaemic–euglycaemic clamp with stable isotopes and to investigate associations between sites of insulin resistance by ethnicity. We hypothesise that in early type 2 diabetes, BAM will have greater hepatic and adipose tissue insulin sensitivity because of their lower VAT deposition compared with their white European counterparts.

## Methods

The study was conducted at the Clinical Research Facility, King’s College London, London, UK and approved by the London Bridge National Research Ethics Committee (12/LO/1859); all participants provided informed consent. The data were collected as part of the South London Diabetes and Ethnicity Phenotyping (Soul-Deep) study; recruitment and data collection took place during the period April 2013 to January 2015 [25, 26].

### Participants

BAM and WEM (self-declared ethnicity, confirmed by grandparental birthplace), aged 18–65 years, BMI 25–35 kg/m^2^, with a diagnosis of type 2 diabetes within 5 years, treated with lifestyle advice ± metformin, with HbA_1c_ ≤63.9 mmol/mol (<8%) were recruited from South London primary care practices and selected to be similar in age and BMI. Participants were deemed ineligible if: treated with thiazolidinedione, insulin, chronic oral steroids, beta-blockers; serum creatinine >150 μmol/l; serum alanine transaminase level >2.5-fold above the upper limit of the reference range; positive auto-antibodies for insulin, GAD or A2; sickle cell disease (trait permitted); or were using medications believed to affect the outcome measures. Participants completed a comprehensive medical screening before study entry.

### Study design

Participants arrived at the Clinical Research Facility in a fasted state, having refrained from eating or drinking anything other than water from 22:00 h the night before. Participants were instructed to refrain from strenuous physical activity in the 48 h preceding the visit, refrain from consuming alcohol in the 24 h preceding the visit and to consume a standardised diet the day prior (~50% of energy from carbohydrate, evenly spread throughout the day, with no more than 30% of daily carbohydrate consumed in the evening meal). Participants using metformin were instructed to stop taking it for 7 days before the visit.

### Hyperinsulinaemic–euglycaemic clamp assessments

On arrival, participants were weighed in light clothing and their body surface area (BSA) calculated using the Mosteller formula. A cannula was inserted into an antecubital fossa vein to infuse stable isotopically labelled tracers, 20% (wt/vol) dextrose and insulin (Actrapid, Novo Nordisk, Bagsvaerd, Denmark) bound to albumin. A second cannula was inserted retrogradely into the dorsum of the hand, which was placed in a hand-warming unit, to achieve arterialised venous blood samples. A baseline blood sample determined the participant’s fasting plasma glucose; if above 5 mmol/l, a sliding scale insulin infusion was used to lower the circulating glucose to 5 mmol/l. At time point −120 min, a primed (2.0 mg/kg), continuous (0.02 mg kg^−1^ min^−1^) infusion of [6,6-^2^H_2_]-glucose and a primed (0.12 mg/kg), continuous (0.0067 mg kg^−1^ min^−1^) infusion of [^2^H_5_]-glycerol (CK Gases, Cambridgeshire, UK) were initiated [27]. Basal state blood samples were taken between −30 and 0 min. After infusion of the tracers for 120 min (basal period), a two-stage hyperinsulinaemic–euglycaemic clamp procedure was started at time point 0 min and continued for 4 h (during which the infusion of [6,6-^2^H_2_]-glucose was continued but the infusion of [^2^H_5_]-glycerol was stopped just prior to beginning the second stage of the insulin infusion). Insulin was infused at a rate of 10 mU m^−2^ BSA min^−1^ (initiated with a priming dose of 30 mU m^−2^ BSA min^−1^ for 3 min and then 20 mU m^−2^ BSA min^−1^ for 4 min) during stage 1 (0–120 min) and at a rate of 40 mU m^−2^ BSA min^−1^ (initiated with a priming dose of 120 mU m^−2^ BSA min^−1^ for 3 min and then 80 mU m^−2^ BSA min^−1^ for 4 min) during stage 2 (120–240 min) [19, 28]. Euglycaemia (5 mmol/l) was maintained by variable infusion of 20% (wt/vol) dextrose, which was enriched with [6,6 ^2^H_2_]-glucose (8 mg/g glucose with low-dose insulin and 10 mg/g with high-dose insulin) to ensure a constant glucose tracer-to-tracee ratio (TTR). Plasma glucose readings were taken every 5 min, using an automated glucose analyser, to inform adjustment of the glucose infusion rate. Hepatic and adipose tissue insulin sensitivity were evaluated in the low-dose insulin infusion, whole body and peripheral (primarily skeletal muscle) insulin sensitivity were evaluated in the high-dose insulin infusion. Blood samples were collected before beginning the tracer infusions to determine baseline enrichment of glucose and glycerol. At time points −30, −20, −10 and 0 min baseline blood samples were collected, followed by sampling at 30, 60, 90, 100, 110, 120, 150, 180, 210, 220, 230 and 240 min for the assessment of plasma glucose and glycerol concentrations and enrichments, insulin and NEFA concentrations.

### Magnetic resonance imaging

Participants attended the magnetic resonance imaging (MRI) unit of Guy’s Hospital, King’s College London, for the assessment of subcutaneous adipose tissue (SAT), visceral adipose tissue (VAT) and skeletal muscle mass. Scanning was performed on a 1.5 T Siemens scanner to acquire magnetic resonance images from the neck to the knee (excluding the arms). Participants lay supine with body coils secured on the scanned body area.

For each participant, the MRI scan produced 320 contiguous axial fat and water images, each 3 mm apart. The Dixon-MRI T1-weighted spin-echo sequence includes an echo time of 4.77 ms for the in-phase images, 2.39 ms for the out-of-phase images and a repetition time of 6.77 ms. Magnetic resonance images were analysed using a semi-automated method carried out by Klarismo (London, UK) to quantify SAT and skeletal muscle mass volumes in all images between the neck and knee region and VAT volume in the whole abdominal cavity.

### Analyses of samples

Plasma glucose concentration was measured by automated glucose analyser (Yellow Spring Instruments, 2300 STAT Glucose Analyzer, Yellow Springs, OH, USA). Serum insulin concentration was measured by immunoassay using chemiluminescent technology (ADVIA Centaur System, Siemens Healthcare, Camberley, UK). Plasma NEFA were measured by an enzymatic colorimetric assay (Wako Diagnostics, Richmond, VA, USA) on an automated clinical chemistry analyser (ILab 650, Instrument Laboratories, Holliston, MA, USA). The glucose and glycerol enrichment (TTR) in plasma were measured by gas chromatography-mass spectrometry on an Agilent GCMS 5975C MSD (Agilent Technologies, Wokingham, UK) using selected ion monitoring. The isotopic enrichment of glucose was determined as the penta-*O*-trimethylsilyl-d-glucose-*O*-methyloxime derivative [29]. The isotopic enrichment of plasma glycerol was determined as the tert-butyl trimethylsilyl (tBDMS) glycerol derivative [30].

### Calculations

Total glucose disposal rate (*M* value in mg kg^−1^ min^−1^) was calculated as a measure of whole body insulin sensitivity. This was computed as the mean of the glucose infusion rate, corrected for any change in measured plasma glucose concentration, during the final 30 min of the high-dose insulin infusion [28]. Additionally, *M* was adjusted for mean insulin concentration (*M*/I).

Peripheral glucose utilisation (glucose rate of disappearance [*R*_d_], μmol kg^−1^ min^−1^), endogenous glucose production (glucose rate of appearance [*R*_a_], μmol kg^−1^ min^−1^) and whole body lipolysis (glycerol *R*_a_, μmol kg^−1^ min^−1^) were calculated using Steele’s non-steady-state equations modified for stable isotopes assuming a volume distribution of 22% body weight [31]. Calculation of glucose kinetics was modified for inclusion of [6,6-^2^H_2_]-glucose in the dextrose infusion [32]. Before calculation of glucose and glycerol kinetics, enrichment and concentrations were smoothed using optical segments analysis [33].

Peripheral glucose utilisation (glucose *R*_d_) was calculated during the basal state and the final 30 min of the high-dose insulin infusion. We used the percentage increase in glucose *R*_d_ from basal to the high-dose insulin infusion as a measure of skeletal muscle insulin sensitivity [34].

Endogenous glucose production (glucose *R*_a_) was calculated by subtracting the exogenous glucose infusion rate from total glucose *R*_a_. Glucose *R*_a_ was calculated during the basal state and during the final 30 min of the low-dose insulin infusion. Percentage suppression of glucose *R*_a_ from basal to the low-dose insulin infusion was calculated as a measure of hepatic insulin sensitivity [35].

Whole body lipolysis (glycerol *R*_a_) was calculated during the basal state and during the final 30 min of the low-dose insulin infusion. Percentage suppression of glycerol *R*_a_ from basal to the low-dose insulin infusion was calculated as a measure of adipose tissue insulin sensitivity [34].

The AUC for plasma glucose, insulin and NEFA concentrations during the clamp were calculated using the trapezium rule.

### Statistics

All variables were checked for normality using the Shapiro–Wilk test and non-normally distributed variables were transformed (log_10_) for analysis. Normally distributed data are expressed as mean (SD), log-normal data were back transformed to give geometric mean and 95% CI and data that remained skewed after log transformation are expressed as median (interquartile range [IQR]). Ethnic differences between means were determined using the independent samples Student’s *t* test for normally distributed data and Mann–Whitney *U* test for skewed data. Mean difference or the ratio of the geometric mean and 95% CI are presented where appropriate. Associations between insulin sensitivity measures and with VAT were tested using Pearson’s correlation coefficient. Multiple regression analyses were conducted to adjust for the effect of body composition (VAT and skeletal muscle mass) on insulin sensitivity measures. Linear regression analysis was used to determine the impact of ethnicity (interaction) on the associations between insulin sensitivity measures. A value of *p* < 0.05 was considered statistically significant. Analyses were performed using SPSS software, version 22 (IBM Analytics, Armonk, NY, USA).

## Results

### Participant characteristics

The clinical characteristics of the participants are shown in Table 1. By design, the groups were not statistically different in age, weight and BMI. Waist circumference, BSA, SAT, number of years following diabetes diagnosis, HbA_1c_ and the proportion of those treated with metformin were not different between ethnic groups. Mean VAT mass was 34.5% lower and mean skeletal muscle mass was 11.9% greater in BAM (Table 1).

**Table 1:** Clinical characteristics of BAM and WEM with type 2 diabetes

Characteristic	BAM	WEM	Sample sizeBAM/WEM	*p*
Age (years)^a^	54.0 (47.9, 60.2)	59.0 (55.5, 62.5)	18/15	0.51
Weight (kg)	90.9 (9.3)	94.2 (11.6)	18/15	0.38
Height (cm)	175.6 (7.6)	176.8 (5.8)	18/15	0.91
BMI (kg/m^2^)	29.5 (2.7)	30.1 (2.7)	18/15	0.55
Waist circumference (cm)	103.6 (8.4)	107.5 (8.8)	18/15	0.20
BSA (m^2^)	2.08 (0.14)	2.13 (0.15)	18/15	0.40
VAT mass (kg)	3.72 (1.07)	5.68 (2.43)	17/14	0.01*
SAT mass (kg)	11.8 (3.9)	11.8 (2.6)	16/14	0.98
Skeletal muscle mass (kg)^b^	20.7 (2.5)	18.5 (3.0)	17/14	0.03*
Duration of diabetes (years)^a^	3.0 (2.5, 3.6)	3.0 (2.0, 4.0)	18/15	0.74
HbA_1c_ (mmol/mol)	50.4 (7.5)	48.6 (7.8)	18/15	0.50
Systolic BP (mmHg)	138.4 (13.6)	131.8 (13.9)	18/15	0.18
Diastolic BP (mmHg)	86.9 (5.1)	82.9 (10.1)	18/15	0.19
Total cholesterol (mmol/l)	4.17 (0.68)	4.30 (0.72)	18/15	0.61
LDL-cholesterol (mmol/l)	2.37 (0.53)	2.29 (0.70)	18/15	0.71
HDL-cholesterol (mmol/l)	1.19 (0.38)	1.24 (0.24)	18/15	0.66
Triacylglycerol (mmol/l)^c^	1.20 (0.95, 1.52)	1.58 (1.26, 1.97)	18/15	0.09
Treated with metformin (%)	78^d^	53^e^	18/15	0.16

Data expressed as mean (SD) for normally distributed data unless otherwise shown

^a^Median (interquartile range) for skewed distributed data

^b^Skeletal muscle mass was measured from neck to knee excluding arms

^c^Geometric mean (95% CI) for log-transformed data or as percentage of individuals where required

^d^*n* = 14

^e^*n* = 8

*p < 0.05

*p* values were generated using an independent sample Student’s *t* test for normally distributed data and Mann–Whitney *U* test for skewed data to compare BAM and WEM

### Whole body, skeletal muscle and hepatic and adipose tissue insulin sensitivity

**Fig. 1:**
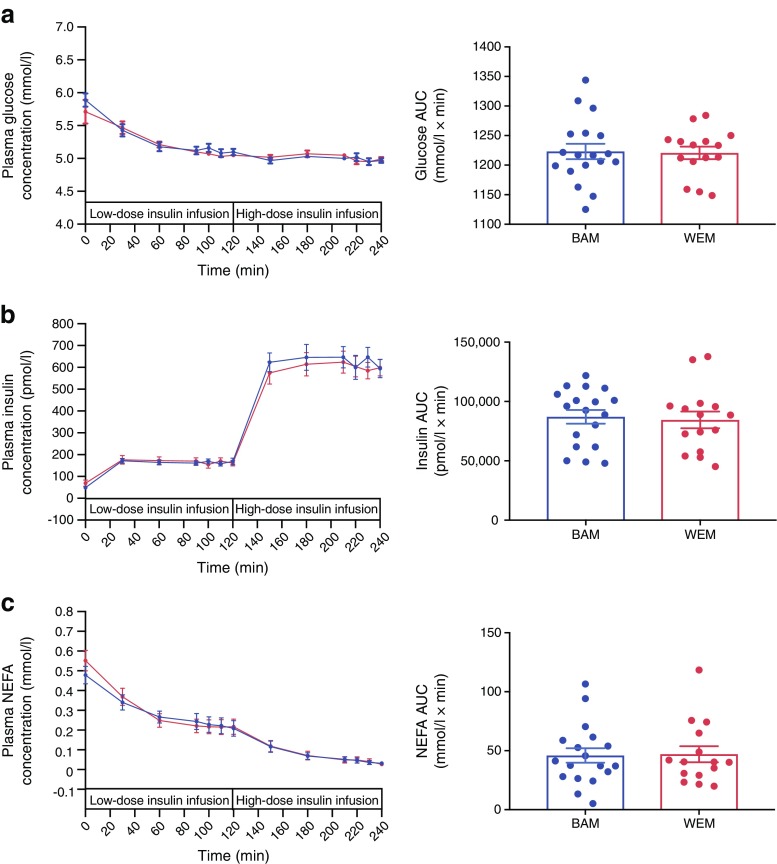
Plasma glucose (**a**), insulin (**b**) and NEFA (**c**) concentrations for BAM and WEM with type 2 diabetes at baseline, and with low-dose and high-dose insulin infusions during the hyperinsulinaemic–euglycaemic clamp. Data are expressed as mean (SEM) for each time point and glucose, insulin and NEFA AUC. Blue circles, BAM; red circles, WEM. Statistical significance between BAM and WEM was assessed using an independent sample Student’s *t* test; there were no significant differences between BAM and WEM

There were no differences in results between the two groups (presented as BAM vs WEM): basal plasma glucose, 5.89 (0.39) vs 5.71 (0.63) mmol/l, *p* = 0.38; insulin, 45.7 (36.8, 56.7) vs 57.3 (39.5, 83.2) pmol/l, *p* = 0.24; and NEFA, 0.48 (0.17) vs 0.55 (0.18) mmol/l, *p* = 0.30. There were no ethnic differences in plasma glucose (*p* = 0.89), insulin (*p* = 0.78) or NEFA (*p* = 0.70) concentrations during the clamp expressed as AUC (Fig. 1). Total glucose disposal rate (*M*), as a measure of whole body insulin sensitivity, did not differ between the ethnic groups (Table 2); the lack of significance continued after adjustment for mean insulin during the high-dose insulin infusion (*M*/I BAM, 0.030 [0.017]) vs WEM, 0.026 [0.011] mg kg^−1^ min^−1^ pmol l^−1^, *p* = 0.46).

**Table 2:** Two-stage hyperinsulinaemic–euglycaemic clamp assessment of insulin sensitivity in BAM and WEM with type 2 diabetes

Measurement	Basal				Hyperinsulinaemic–euglycaemic clamp			
	BAM *n* = 15	WEM *n* = 12	Mean difference or ratio of the geometric mean (95% CI) (BAM − WEM) (95% CI)	*p*	BAM *n* = 18	WEM *n* = 15	Mean difference (BAM − WEM) (95% CI)	*p*
Glucose disposal rate (*M*; mg kg^−1^ min^−1^)	–	–	–	–	4.52 (2.07)	4.00 (1.70)	0.52 (−0.82, 1.89)	0.44
Peripheral glucose utilisation (*R*_d_; μmol kg^−1^ min^−1^)	–	–	–	–	26.8 (10.4)	24.2 (8.5)	2.60 (−4.22, 9.41)	0.44
Endogenous glucose production (*R*_a_; μmol kg^−1^ min^−1^)	8.82 (1.49)	9.25 (1.66)	−0.43 (−1.69, 0.81)	0.48	5.76 (1.73)	6.50 (2.34)	−0.74 (−2.18, 0.71)	0.31
Lipolysis (glycerol *R*_a_; μmol kg^−1^ min^−1^)	1.51 (1.31, 1.75)	1.82 (1.55, 2.15)	0.83 (0.67, 1.02)	0.08	1.06 (0.47)	1.18 (0.33)^a^	−0.12 (−0.43, 0.19)	0.43

Data expressed as mean (SD) for normally distributed data and geometric mean (95% CI) for skewed data

*M* values and glucose *R*_d_ assessments were derived from the high-dose insulin infusion (40 mU m^−2^ BSA min^−1^), glucose and glycerol *R*_a_ assessments were derived from the low-dose insulin infusion (10 mU m^−2^ BSA min^−1^) of the hyperinsulinaemic–euglycaemic clamp and at baseline

^a^WEM sample size = 13

*p* values were generated using an independent sample Student’s *t* test to compare BAM and WEM

**Fig. 2:**
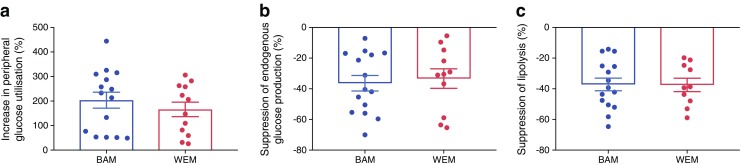
Insulin-mediated peripheral glucose uptake (**a**), suppression of endogenous glucose production (**b**) and suppression of lipolysis (**c**), calculated as percentage change from basal to the low- or high-dose insulin infusion. Differences in sample size from Table 2 are due to a small number of participants missing basal data owing to the administration of a sliding scale insulin infusion to achieve euglycaemia 5 mmol/l prior to beginning the clamp. Data are expressed as mean (SEM). Statistical significance between BAM and WEM was assessed using an independent sample Student’s *t* test; there were no significant differences between BAM and WEM

Peripheral glucose utilisation (glucose *R*_d_) during the high-dose insulin infusion (Table 2) and skeletal muscle insulin sensitivity (% increase in peripheral glucose utilisation) were also similar between ethnic groups (Fig. 2a; BAM, 203.5% [126.2] vs WEM, 166.3% [102.5], mean difference of 37.3%, 95% CI −55.6, 130.1; *p* = 0.42). Basal endogenous glucose production (glucose *R*_a_) was similar between BAM and WEM and there were no ethnic differences in endogenous glucose production during the low-dose insulin infusion (Table 2) or in hepatic insulin sensitivity (% suppression of endogenous glucose production) (Fig. 2b; BAM, −36.4% [19.7] vs WEM, −34.8% [20.7], mean difference of −1.61%, 95% CI −17.7, 14.5; *p* = 0.84).

There was no significant difference in basal whole body lipolysis (glycerol *R*_a_) in BAM compared with WEM (*p* = 0.08; Table 2). There were no ethnic differences in lipolysis during the low-dose insulin infusion or in adipose tissue insulin sensitivity (% suppression of lipolysis) (Fig. 2c; BAM, −37.2% [16.0] vs WEM, −37.5% [13.7], mean difference of 0.32%, 95% CI −12.5, 13.1; *p* = 0.96). After adjustment for VAT and skeletal muscle mass, we found no ethnic differences in whole body insulin sensitivity (mean difference, 0.95 mg kg^−1^ min^−1^, 95% CI −0.48, 2.37; *p* = 0.18) or skeletal muscle insulin sensitivity (mean difference, 82.3%, 95% CI −23.1, 187.8; *p* = 0.12). Similarly when we adjusted hepatic and adipose tissue insulin sensitivity for VAT we found no ethnic differences, with a mean difference of 9.1%, 95% CI −9.8, 28.0, *p* = 0.33 and 8.4%, 95% CI −5.7, 22.4, *p* = 0.23, respectively. Correlation analysis of hepatic insulin sensitivity with VAT was only significant in BAM (BAM, *r* = −0.55, *p* = 0.04; WEM, *r* = −0.23, *p* = 0.50) and when we correlated VAT with adipose tissue insulin sensitivity we found no association in BAM (*r* = −0.13, *p* = 0.66) or in WEM (*r* = −0.60, *p* = 0.09).

### Associations between tissue-specific sites of insulin sensitivity

**Fig. 3:**
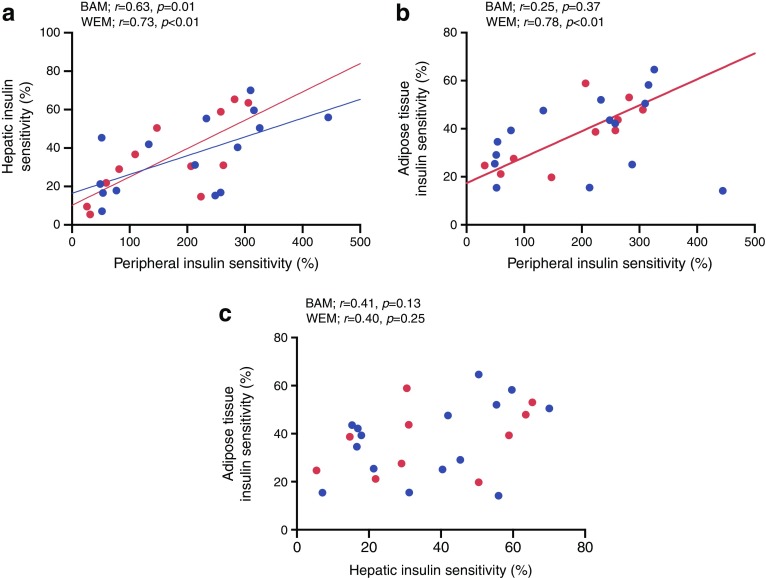
Associations between tissue-specific insulin sensitivities during the hyperinsulinaemic–euglycaemic clamp in BAM and WEM with early type 2 diabetes: (**a**) peripheral (calculated as the percentage increase in glucose *R*_d_ from basal to high-dose insulin infusion, 40 mU m^−2^ BSA min^−1^) and hepatic insulin sensitivity (calculated as the percentage suppression of glucose *R*_a_ from basal to low-dose insulin infusion, 10 mU m^−2^ BSA min^−1^); (**b**) peripheral and adipose tissue (calculated as the percentage suppression of glycerol *R*_a_ from basal to low-dose insulin infusion, 10 mU m^−2^ BSA min^−1^) insulin sensitivity; and (**c**) hepatic and adipose tissue insulin sensitivity. Data expressed using Pearson’s correlation coefficient with corresponding *p* values for BAM and WEM. Sample size: BAM, *n* = 15; WEM, *n* = 12 (except for adipose tissue insulin sensitivity analyses, where *n* = 10 for WEM). Blue dots and regression line, BAM; red dots and regression line, WEM

As shown in Fig. 3a, we found a significant correlation between skeletal muscle (% increase in *R*_d_) and hepatic insulin sensitivity (% suppression of *R*_a_) in both ethnicities. We found no significant correlation between skeletal muscle and adipose tissue insulin sensitivity (% suppression of glycerol *R*_a_) in BAM; however, there was a strong correlation in WEM (Fig. 3b). There were no significant correlations between hepatic and adipose tissue insulin sensitivity in either ethnicity (Fig. 3c). We further explored the impact of ethnicity on these associations using regression analysis and found no significant ethnicity interaction for the impact of hepatic tissue insulin sensitivity on skeletal muscle insulin sensitivity (*p* = 0.82), adipose tissue insulin sensitivity on skeletal muscle insulin sensitivity (*p* = 0.26) or adipose tissue insulin sensitivity on hepatic insulin sensitivity (*p* = 0.84).

## Discussion

To our knowledge this is the most comprehensive comparison of whole body, skeletal muscle, hepatic and adipose tissue insulin sensitivity in a single study between adults of black African and white European ethnicity with early type 2 diabetes. We have found, in BAM and WEM with early type 2 diabetes and of similar BMI and age, comparable whole body, skeletal muscle, hepatic and adipose tissue insulin sensitivity despite lower VAT deposition and greater skeletal muscle mass in BAM. In addition, we have shown ethnic differences in the associations between tissue-specific sites of insulin sensitivity, which adds to the concept of ethnic distinctions in type 2 diabetes pathophysiology.

Lower visceral fat deposition has been extensively reported in black populations compared with other ethnic groups [5] and we hypothesised that, consequently, BAM would exhibit greater adipose tissue and hepatic insulin sensitivity. Although our data did not show a significant relationship between adipose insulin sensitivity and VAT, which may be due to the sample size, we did detect lower VAT and a possible, but non-significant, trend towards lower basal lipolysis in our BAM group, which agrees with the majority of the literature. However, we did not find greater adipose tissue insulin sensitivity (even after adjustment for VAT), which contrasts with the findings of a study in black women [14], notably this study used a lower insulin dose than ours and the women were free of type 2 diabetes, which may explain some of the inconsistencies between our findings. Our men had recently been diagnosed with type 2 diabetes, thus pathophysiological changes may be present that are not seen in non-diabetic groups. Likewise, the presence of diabetes may be important in the lack of greater hepatic insulin sensitivity in our black men, as per our hypothesis. Our findings agree with studies in adolescents [7, 10–13, 20, 23] and a single study in lean non-diabetic women [18] but do not agree with studies in obese non-diabetic women [17, 19] suggesting that, in addition to glycaemic status, body composition may also play a role in the ethnic comparison. We did detect ethnic differences in the association between VAT and hepatic insulin sensitivity however our results were consistent after adjustment for VAT, hence future studies controlling for other ectopic fat depots could help us to understand the impact of adiposity and ethnicity in type 2 diabetes.

Another finding from this study is the lack of ethnic differences in whole body or skeletal muscle insulin sensitivity, which remained even after we adjusted for differences in VAT and skeletal muscle mass. An extensive literature base exists in which black populations are noted to exhibit pronounced insulin resistance compared with other ethnic groups; however, the majority of these studies have used methods which estimate, rather than directly measure, insulin sensitivity [36] and even in studies using the hyperinsulinaemic–euglycaemic clamp method, mixed results are reported [37]. Again, the type 2 diabetes status of our participants is important here, as the presence of diabetes may have attenuated any pre-morbid ethnic differences in insulin sensitivity. This suggestion is supported by the results from a large study of diabetic and non-diabetic populations [38] in which the intravenous glucose tolerance test was used to assess insulin sensitivity. While ethnic differences were present in the non-diabetic state [38], they were absent in type 2 diabetes [39], suggesting that by the end of the glucose tolerance spectrum ethnic differences in insulin sensitivity may have dissipated. It is also reasonable to propose that the adiposity status of our participants may explain the absence of ethnic differences in insulin sensitivity. We aimed to achieve a similar mean body mass index in our two ethnic groups. Their body weights were, on average, in the overweight and obese range, as is typical for people with type 2 diabetes, hence the impact of excess adiposity may also have attenuated any ethnic differences, as discussed in other studies comparing ethnicity in populations with type 2 diabetes [23]. However, insulin sensitivity data, which have been stratified for obese and non-obese in type 2 diabetes, have also shown no ethnic differences [39] suggesting that our result is real and driven more by the presence of type 2 diabetes. In addition to obesity status, we were able to assess skeletal muscle mass which was found to be higher in BAM. Having the same whole body and skeletal muscle insulin sensitivity in the presence of greater skeletal muscle mass, and having the same hepatic and adipose sensitivity in the presence of reduced VAT, suggests that the BAM may be more insulin resistant when adjusted for lean mass [23, 40]; however, we did not find this and there may be other confounding factors, such as muscle and hepatic lipid content, that explain this finding but that are not investigated here.

Increased NEFA release (lipolysis), which occurs during excess adiposity, particularly visceral adiposity, has been shown to impair glucose homeostasis through the process of lipotoxicity. The NEFAs impair insulin signalling and lead to skeletal and hepatic insulin resistance, contributing to the pathophysiology of type 2 diabetes [41–43]. We would therefore expect to see a significant relationship between lipolysis and both skeletal muscle and hepatic insulin sensitivity. Our data show a strong association between lipolysis and skeletal muscle insulin sensitivity in WEM that was not present in BAM. This may suggest an independent relationship of adipose tissue lipolysis with skeletal muscle glucose uptake in BAM and may imply that mechanisms other than lipotoxicity are central to the development of hyperglycaemia in BAM. We do, however, acknowledge that our regression analysis failed to support an impact of ethnicity on the relationship between lipolysis and skeletal muscle sensitivity, which may result from the small sample size in our study. Although we have not directly measured lipotoxicity, as this involves a combination of increased NEFA availability and uptake into the muscle, the concept of an independent relationship between glucose and lipid metabolism is supported by a number of studies that have identified the presence of hyperglycaemia in the absence of pronounced ectopic fat, particularly visceral fat [26]. Further investigation of muscle lipid uptake, insulin signalling and ectopic fat deposition would help to improve our understanding of the impact of lipotoxicity on skeletal muscle insulin resistance in black populations.

The strengths of this study lie in our use of the hyperinsulinaemic–euglycaemic clamp with stable isotope infusions to directly assess and compare tissue-specific insulin sensitivity in vivo in a single study [28]. In particular, the use of a glycerol tracer as opposed to a NEFA tracer allows for a direct measure of NEFA release because, unlike NEFA, glycerol is not recycled back into triacylglycerol [44]. Using a two-stage hyperinsulinaemic–euglycaemic clamp allowed for a low- and high-dose insulin infusion to be applied, enabling quantification of suppression of endogenous glucose production and lipolysis, which is missed when only a high-dose insulin infusion is used [45]. All of the studies assessing hepatic and skeletal insulin sensitivity in adolescents have used a single high-dose insulin clamp in which endogenous glucose production is near maximally suppressed, preventing assessment of suppression of endogenous glucose production. Our study is necessarily small because of the complexity of our protocol; however, it is comparable with other studies using these methods in type 2 diabetes [23, 24]. Furthermore, these data were collected as part of a larger study powered to investigate ethnic differences in beta cell function (reported elsewhere by Mohandas et al [26]) and we acknowledge that our sample size may hinder the conclusions we can draw from these data. While we made an effort to control the dietary intake prior to the metabolic assessments, which may have impacted on metabolism and insulin sensitivity, we did not undertake a formal analysis of adherence to this aspect of the protocol. We also must consider that the insulin dosage we used in our high-dose stage may not have been sufficient to induce full suppression of endogenous glucose production for our most insulin-resistant participants; however, on average we achieved 80% suppression from the basal level.

In conclusion, we have found that in early type 2 diabetes there are no ethnic differences in insulin sensitivity between BAM and WEM despite BAM having lower visceral fat and higher skeletal muscle mass. While adipose tissue lipolysis is strongly associated with skeletal muscle insulin sensitivity in WEM, there is less evidence for an association in BAM, suggesting an independent relationship between glucose and lipid metabolism may exist within the development of type 2 diabetes in this ethnic group.

## Data Availability

The data that support the findings of this study are available from the corresponding author upon reasonable request.

## Abbreviations

BSA: Body surface area
BAM: Black (West) African men
MRI: Magnetic resonance imaging
*R*_a_: Rate of appearance
*R*_d_: Rate of disappearance
SAT: Subcutaneous adipose tissue
TTR: Tracer-to-tracee ratio
VAT: Visceral adipose tissue
WEM: White European men
